# Grapevine–Downy Mildew Rendezvous: Proteome Analysis of the First Hours of an Incompatible Interaction

**DOI:** 10.3390/plants9111498

**Published:** 2020-11-05

**Authors:** Rita B. Santos, Rui Nascimento, Ana V. Coelho, Andreia Figueiredo

**Affiliations:** 1 Biosystems & Integrative Sciences Institute (BioISI), Faculdade de Ciências, Universidade de Lisboa, Campo Grande, 1749-016 Lisboa, Portugal; absantos@fc.ul.pt (R.B.S.); rfnascimento@fc.ul.pt (R.N.); 2 Instituto de Tecnologia Química e Biológica, Universidade Nova de Lisboa, Av. da República, 2780-157 Oeiras, Portugal; varela@itqb.unl.pt

**Keywords:** *Vitis vinifera*, *Plasmopara viticola*, plant–pathogen interaction, proteomics, defense-related proteins, ETS, ETI

## Abstract

Grapevine is one of the most relevant crops in the world being used for economically important products such as wine. However, relevant grapevine cultivars are heavily affected by diseases such as the downy mildew disease caused by *Plasmopara viticola*. Improvements on grapevine resistance are made mainly by breeding techniques where resistance traits are introgressed into cultivars with desired grape characteristics. However, there is still a lack of knowledge on how resistant or tolerant cultivars tackle the *P. viticola* pathogen. In this study, using a shotgun proteomics LC-MS/MS approach, we unravel the protein modulation of a highly tolerant grapevine cultivar, *Vitis vinifera* “Regent”, in the first hours post inoculation (hpi) with *P. viticola*. At 6 hpi, proteins related to defense and to response to stimuli are negatively modulated while at 12 hpi there is an accumulation of proteins belonging to both categories. The co-occurrence of indicators of effector-triggered susceptibility (ETS) and effector-triggered immunity (ETI) is detected at both time-points, showing that these defense processes present high plasticity. The results obtained in this study unravel the tolerant grapevine defense strategy towards *P. viticola* and may provide valuable insights on resistance associated candidates and mechanisms, which may play an important role in the definition of new strategies for breeding approaches.

## 1. Introduction

Plant immunity is an intricate system that involves a remarkable array of structural, chemical, and protein-based layers of defense aimed at stopping pathogens before they cause irreversible damages. When pathogens overcome these first barriers, namely cuticle, cell wall, hairs, or antimicrobial compounds, they elicit an elaborate recognition system that culminates with the activation of broad-range plant defenses [1,2]. Plant transmembrane pattern-recognition receptors (PRRs) recognize conserved molecules (pathogen-associated molecular patterns, (PAMPs)), activating an immune response leading to the establishment of PAMP-triggered immunity (PTI). PTI is often associated with the production of reactive oxygen species (ROS), activation of mitogen-activated protein kinase (MAPK) cascades and transcriptional induction of defense-related genes [3,4]. This front-line defense must be overcome by pathogens for a successful colonization [5]. If virulence determinants, i.e., effector molecules, are able to suppress immunity through direct molecular interactions with host defense-associated proteins, effector-triggered susceptibility (ETS) is established, reflecting the co-evolution between host and pathogen [6,7]. To counterattack this, plants have evolved to produce resistance proteins (R proteins) that recognize specific pathogen effector molecules. This recognition starts effector triggered immunity (ETI) and a broader modulation of plant defense system occurs, often accompanied by programmed cell death and pathogen restrain [5].

Grapevine (*Vitis vinifera* L.) represents a great agricultural and economic value worldwide, with deep ties to human culture for more than 5000 years. In 2019, grapevine plantation areas reached 7.4 Mha, with Spain, Italy, and France leading these plantation areas in Europe [8]. The European elite grapevine cultivars are highly susceptible to various pathogens. The obligatory biotrophic oomycete *Plasmopara viticola* (Berk. & M.A. Curtis) Berl. & De Toni, the causative agent of grapevine downy mildew, is one of the most devastating [9,10]. This pathogen was introduced into European vineyards in the 1870s and quickly spread to the world’s major grape-producing regions [10]. *Plasmopara viticola* infects all the green parts of the plant, leading inevitably to major yield losses [10]. Preventive application of fungicides is the common strategy to control the downy mildew disease, and represents almost two thirds of all synthetic fungicides sprayed in the European Union with adverse effects on the environment. This approach is proving to be progressively less effective as recent reports have shown that several *P. viticola* isolates were able to develop fungicide resistance [11,12]. Moreover, there is an increasing demand for more sustainable agricultural practices, with several guidelines being established within the European Union (Directive 2009/128/EC). Over the last decades, breeding for resistance has been implemented as an alternative to the use of fungicides. This process aims to obtain new cultivars with pathogen resistance without impacting other desirable traits [13]. Partially resistant grapevine crossing hybrids are already in the market, e.g., “Regent”, “Solaris”, and “Bianca”. These hybrids result from breeding programs where introgression of resistance traits from highly tolerant wild American and Asian *Vitis* spp. (e.g., *Vitis labrusca* and *Vitis amurensis*) was promoted. However, *P. viticola* presents a strong evolutionary potential as several isolates have been able to break down interspecific hybrid plant resistance [11,14]. These observations highlight the need for a deeper understanding on plant immunity, particularly on the establishment of the grapevine–*P. viticola* incompatible interaction.

Various studies have shown that *P. viticola* resistance is based primarily on post-infection processes, apart from physical and chemical barriers [15,16] and that the establishment of an effective defense response depends on a recognition system specific to *P. viticola* [17]. Thus, unveiling grapevine response during the first h of pathogen challenge is crucial for a deeper understanding of pathogen recognition and defense activation mechanisms. Although scarce, proteomic studies have opened new insights, particularly on the establishment of the compatible interaction (reviewed in [18]). A transient breakdown in defense responses was reported in susceptible genotypes, although a high abundance of defense and allergenic-related proteins was detected [19,20,21]. A clear difference between pre- and post-infection stages was reported when comparing protein modulation in compatible and incompatible interactions [22,23]. The incompatible interaction in Rpv1 and Rpv3 pyramided resistance loci genotypes is characterized by a high accumulation of redox, energy and defense response proteins [24]. In previous studies, we have characterized the genotype-specific responses to *P. viticola* inoculation by direct comparison of a tolerant (Rpv3.1 background) with a susceptible grapevine genotype using a 2D-DIGE approach. We have shown that the involvement of ROS-signalling events that restrain fungal growth and lipid associated signalling discriminate between both genotypes. In the present study, we investigate quantitative and qualitative modulation of *V. vinifera* cv. Regent proteome, on the first hours of interaction with *P. viticola*, by a high throughput LC-MS/MS analysis. We identified 899 proteins, mainly related to response to stimulus, signalling, cellular organization, and protein metabolism. Overall, our data provide, for the first time, new insights into the molecular processes occurring in the first hours of the incompatible interaction between grapevine and *P. viticola*.

## 2. Results and Discussion

### 2.1. Proteomic Modulation in Grapevine Leaves in the First Hours after P. viticola Inoculation

**Figure 1:**
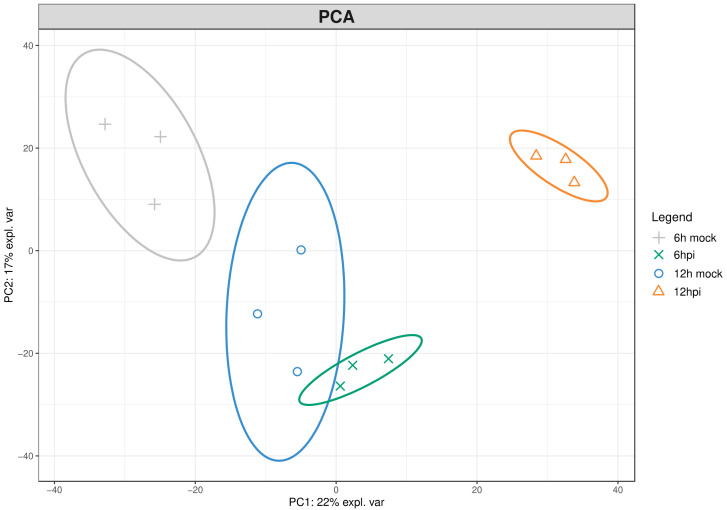
Principal component analysis of the differential protein profiles in *Vitis vinifera* cv ‘Regent’ at 6 and 12 h post-inoculation with *Plasmopara viticola*. Principal component 1 (PC1) on X axis explains 22% of protein variability and principal component 2 (PC2) on Y axis explains 17% of the selected proteins variability. explaining variants (expl. var); hours post inoculation (hpi).

To investigate the first h of the incompatible interaction between *Vitis vinifera* cv. Regent and *Plasmopara viticola*, the leaf proteome was characterized using an untargeted proteomics approach. The proteomic dataset identified 899 proteins (Table S1). Principal components analysis (PCA), a multivariate data analysis method, was used to access the differences among inoculated and control samples at 6 h and 12 h post-inoculation as well as to determine sample associated variations. This unsupervised method allows separation of the variables into principal components whose correspondence to putative biological processes can be assumed without prior biological hypothesis [25]. This approach confirmed a distinction between the proteome of inoculated and control samples (Figure 1).

This unsupervised PCA bi-plot, explains 22% (PC1) and 17% (PC2) of proteome variability (Figure 1), in addition, the clustering of biological replicates within the PCA plots indicates the absence of unwanted variation in the dataset. This increases confidence in the reproducibility of the differential accumulation analysis.

**Figure 2:**
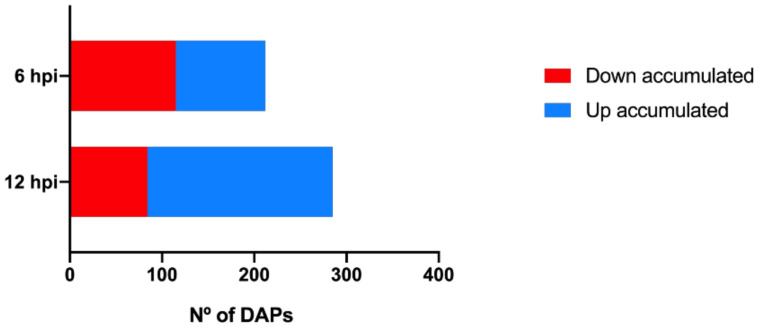
Number of differentially accumulated proteins at each inoculation time-point. Red bars: down accumulated proteins; blue bars: up accumulated proteins; differentially accumulated proteins (DAPs); hours post inoculation (hpi).

Statistical analysis showed that the accumulation of 497 proteins was significantly altered during the first h of pathogen challenge. At 6 h post-inoculation (hpi), 212 differentially accumulated proteins (DAPs) were detected, and 285 proteins were significantly altered at 12 hpi (Figure 2, Tables S2 and S3). Of these, 46% of the proteins were up accumulated at 6 hpi while at 12 hpi this number increased to 71%. The increasing number of infection responsive proteins during inoculation suggests that, although larger transcriptional changes occur as soon as 6 hpi [26], a larger proteome modulation occurs with the growing contact between pathogen and plant. In fact, other studies also report an increase number of *P. viticola* responsive proteins along the inoculation time-course [23,24]. Eighty-four of the identified proteins were differentially accumulated at both time-points.

**Figure 3:**
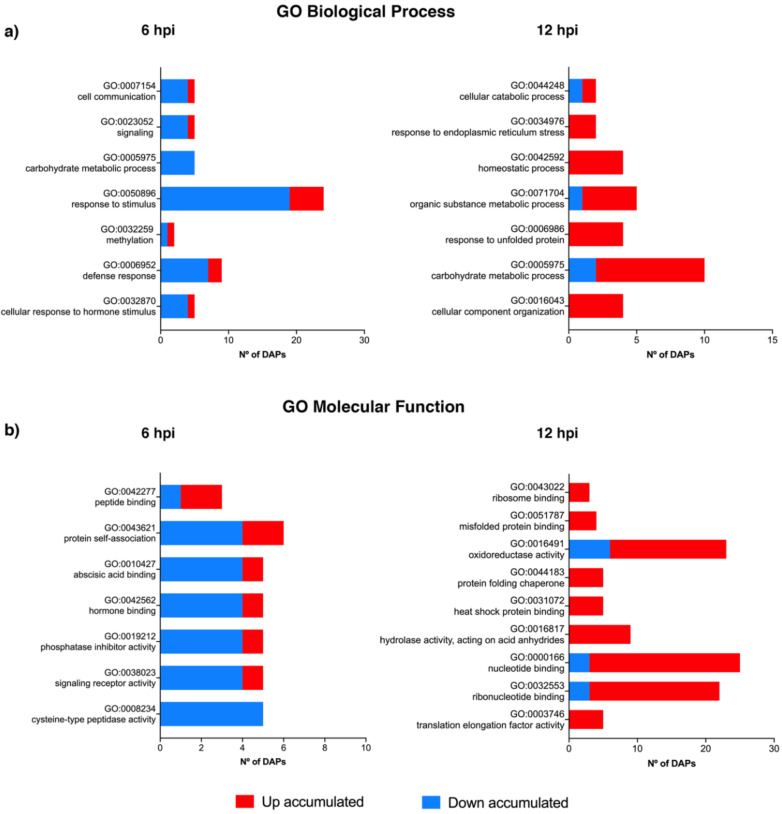
Gene Ontology (GO) enrichment of (**a**) GO Biological Processes terms and (**b**) GO Molecular Function terms of *Vitis vinifera* cv ‘Regent’ proteins at 6 and 12 hpi with *Plasmopara viticola*.: differentially accumulated proteins (DAPs); hours post inoculation (hpi).

A Gene Ontology (GO) enrichment analysis was conducted to access which biological processes and molecular functions undergo significant changes at 6 and 12 hpi (Figure 3). At 6 hpi, the biological processes being significantly repressed are associated to cell communication, signalling, carbohydrate metabolic processes, response to stimulus and defense response (Figure 3; Table S2). Concerning the molecular functions, the activity of cysteine-type peptidases, signalling receptors, phosphate inhibitors, and hormone and abscisic acid binding are negatively modulated. At 12 hpi, most of the biological processes and molecular processes are up-regulated. Biological processes such as response to endoplasmic reticulum (ER) stress, homeostatic process, response to unfolded protein and cellular component organization and molecular functions such as ribosome binding, misfolded protein binding, protein folding chaperone, heat shock protein binding, hydrolase activity and translation elongation factor activity are positively modulated. Although no significant GO term related to defense response was observed in the GO enrichment analysis at 12 hpi, several DAPs detected are related to processes such as innate immune response and regulation of defense response to fungus (incompatible interaction) (Figure 3).

For susceptible genotypes, a time-dependent biphasic modulation has been described, with a clear decline of defense-associated proteins at 48 hpi indicating that *P. viticola* achieved colonization by suppressing the host defense responses [20]. For the incompatible interaction between *P. viticola* in a Rpv1/Rpv3 pyramid resistant grapevine background, it was suggested that no biphasic modulation of defense responses occur after 24 hpi [24]. Although the present study was conducted in the first h of interaction, there is no evidence of a biphasic modulation in defense responses. At 6 hpi the main categories modulated were related to defense and at 12 hpi, despite the main enriched pathways were associated to protein refolding, categories such as response to endoplasmic reticulum stress response, oxireductase activity and hydrolase activity are mostly represented.

When analyzing DAPs in *V. vinifera* leaves upon *P. viticola* inoculation, it was observed that several DAPs are common to both time points. We have further focused our analysis on these common proteins.

### 2.2. Translation and Protein Transport Processes Are Initially Boosted but Become Repressed at 12 hpi

Ribosomal proteins (RPLs) are constituents of the ribosome machinery and are required for the synthesis of proteins. In plant response to stress, RPLs and their coding genes were also pointed out as having an important role [27,28]. The 50S ribosomal protein L3 (RPL3) is one of the first of this group to be recruited into ribosome assembly and it is required for the activity of the peptidyltransferase center, which carries out peptide bond formation, protein elongation, and peptide release [29]. In a study with rice plants, RPL expression was reported to be repressed upon inoculation with dwarf virus [30]. In this study, similar patterns were observed regarding protein accumulation in *V. vinifera* cv Regent upon *P. viticola* challenge (see Table 1). RPL accumulation increased after pathogen inoculation at 6 hpi, decreasing to negative folds at 12 hpi. This suggests that this is one of the processes that may lead to the resistance of Regent towards *P. viticola* infection in the first h of contact. This increased accumulation of ribosome constituent proteins in the first hours of plant–pathogen contact has been reported for several pathosystems to have a potential role in hypersensitive response (HR) response, and thus plant defense.

Two subunits of the nascent polypeptide-associated complex (NAC) were identified to be up accumulated in the Regent cultivar at 6 hpi. This complex binds to eukaryotic ribosomes and promotes translation and protein folding. When there is a disruption in proteostasis balance, such as in cellular stress response, this complex detaches from ribosomes and forms protein aggregates so it can work as a chaperone. In this state, the capacity of transcription decreases as well as the flux of emerging peptides [31]. The two subunits, NAC subunit alpha-like protein 1 and 2, were up accumulated by 3.4 and 1.3-fold respectively in Regent at 6 hpi, presenting negative fold values at 12 hpi. Elagamey and colleagues have described an upregulation of NAC subunit alpha mRNA levels in the early stages of pathogen attack in the wilt (*Fusarium oxysporum*)–chickpea interaction. Moreover, this was only observed when a wilt resistant chickpea cultivar was used [32]. In contrast, a decrease in accumulation of this proteins may indicate a general repression of translation under stress [33].

Apart from proteins related to translation and protein transport, a plant defense-related protein was identified at both time-points: PREDICTED: major allergen Pru ar 1 (*Vitis vinifera*), a protein belonging to the pathogenesis-related protein 10 (PR-10) family. This protein was threefold accumulated in grapevine leaves at 6 hpi and its accumulation decreased to −1.5-fold at 12 hpi. This may indicate that Pru ar 1 is involved in the first line of grapevine defense, in the very first h of plant–pathogen contact. This protein has been described to be induced in a highly resistant soybean cultivar upon infection with the oomycete *Phytophthora sojae* [34]. Lemaître-Guillier and colleagues have also described the increased accumulation (~1.2-fold) of major allergen Pru ar 1 in grapevine leaves upon elicitor treatment and inoculation with *P. viticola* [35]. It has also been shown that this protein is induced in plants affected by different pathogens such as viruses, fungi, and bacteria; thus, it is not pathogen specific. It may act by concentrating at pathogen’s entry sites, creating a protective barrier and increasing plant’s resistance [34].

Finally, a serine/threonine kinase-like protein was also identified in the proteomic analysis. This protein was up accumulated by 1.5-fold at 6 hpi but its accumulation was significantly affected at 12 hpi (−2.1-fold). Serine/threonine kinase genes have been described to have a constitutive and high-level expression in resistant non-inoculated plants, in contrast to susceptible plants where no expression is detected [36]. It has also been suggested that this protein may be involved in boosting resistance against plant pathogens, since they are involved in signalling the presence of the pathogen in the plant [37,38]. A serine/threonine protein kinase from *Arabidopsis thaliana* has been described to be an activator of abscisic acid signalling pathway, regulating numerous responses such as stomata closure in response to plant pathogens among other stresses [39]. These proteins are required for stomata closure and are mediated by PAMPs. They limit the entrance of pathogens by closing the stomata transiently but pathogens have been described to counterattack this by promoting stomata opening [39]. This is in line with the results presented in Table 1, where the accumulation of this protein reduces after 12 h of pathogen inoculation. Since *V. vinifera* Regent hybrid shows only tolerant towards *P. viticola* infection, the pathogen may be forcing stomata opening at 12 hpi in order to complete a successful infection.

### 2.3. Stress Related Proteins Are Negatively Affected at 6 hpi but Accumulated at 12 hpi

Table 2 represents the DAPs that were down accumulated at 6 hpi and up accumulated at 12 hpi. Among those, there was a high representation of proteins related to stress and plant defense.

The accumulation of the aminomethyltransferase protein increased significantly at 12 hpi to 5.4-fold. This has already been described in *V. vinifera* plants at 48 h upon elicitation with two forms of laminarin, which is known to elicit defense responses and induce resistance to *P. viticola* [35]. In this study, the increase in accumulation was observed much earlier, which might be due to a more effective defense response to the presence of the pathogen in contrast to the elicitor.

The elongation factor 1-alfa (EF1α), that was repressed at 6 hpi when compared to the mock sample, was observed to be more accumulated at 12 hpi by 2.6-fold. It has been demonstrated in rice that a EF1α gene is involved in PCD and defense responses [40]. Wang and colleagues showed that a EF1α-like gene has a negative effect in salicylic and jasmonic acid pathways and that its loss of function or suppression leads to an increase in salicylic and jasmonic acid content, thus enhancing the plant’s defense network [40]. This may be the reason why at 6 hpi the protein is down accumulated, indicating that at the first hours of plant–pathogen interaction, Regent grapevine plants boost their network defense to quickly counterattack the *P. viticola* pathogen.

Regarding the actin-depolymerizing factor 2 (ADF2), which was strongly repressed at 6 hpi, its accumulation increased to approximately 3-fold at 12 hpi. This protein has been described to be involved in the plant defense against oomycetes, among other pathogens. In wheat plants silenced for the ADF2 gene, the resistance to the stripe rust fungi (*Puccinia striiformis*) increased by increasing the accumulation of ROS species and by HR. Tang and colleagues also observed that this silencing inhibited pathogen penetration [41]. In *V. vinifera* Regent the same process might be occurring. The strong repression of this protein at 6 hpi might have the function of impeding *P. viticola* penetration in leaf cells and thus increase disease resistance.

A serine hydroxymethyltransferase (SHMT) was also found to be differentially accumulated at both time-points. At 6 hpi, it was down accumulated and its accumulation increased at 12 hpi to 2.3-fold. It has been described that *A. thaliana* plants with a *shmt1-1* mutation are more susceptible to biotrophic and necrotrophic pathogens in relation to control plants [42]. This protein has also been found to increase in effector-triggered immunity (ETI) interactions [43], which is in agreement with the observed data in this study. *V. vinifera* Regent is a tolerant plant to *P. viticola* pathogen and at 12 hpi an ETI response might be taking place in response to pathogen effectors.

Heat sock proteins (HSP) are key players in de novo protein synthesis and in protein translocation to organelles. They are also responsible for the degradation of flawed proteins and protection during stresses. An effector from *P. syringae* (HpI1) has been described to target heat shock 70 kDa protein (HSP70) and hijack it to the chloroplasts, forming a large complex that leads to plant defense impairment by suppression of salicylic acid accumulation [44,45]. In contrast, in the infection of sunflowers with powdery mildew, HSP70 is more highly expressed and accumulated in resistant that in susceptible genotypes [46]. Depending on the plant pathosystem, the role of HSP70 may help the plant to counterattack the pathogen or contribute to weaken the plant defenses. In Regent, this protein accumulated more highly at 12 hpi. In previous studies, we have shown by 2-DIGE that a HSP70 isoform 2 is present, at 6 hpi, in higher abundance in Regent than in “Trincadeira”, a Portuguese susceptible elite cultivar [23]. Together, these studies indicate that HSP70 is more accumulated in resistant than in susceptible grapevine cultivars and that HSP70 isoforms may have a redundant role in grapevine plant defense.

Glyceraldehyde-3-phosphate dehydrogenase B (GAPDHb), a glycolysis related protein, is an enzyme that plays fundamental roles in cell pathways and in adjustment to stress [47]. In plant–pathogen interaction it is secreted to the cell wall in order to strengthen it and to reduce polysaccharide metabolism during pathogen entry [48]. It has also been described that GAPDHb is a target of plant virus proteins. In a yeast two-hybrid screening, this protein interacted with AV2 protein [49]. It has also been shown for bamboo mosaic virus and tobacco bushy stunt virus that viral proteins bind to GADPH to facilitate the viral RNA replication [50,51]. We earlier reported the up regulation of GAPDHa in Regent at 6 hpi, when compared with Trincadeira [23]. The glucose breakdown by this enzyme renders needed energy to aid plants in development processes and immune responses.

### 2.4. Several DAPs Are Significantly Repressed at Both Time Points

Several DAPs were found to be down accumulated at both time-points (Table 3). These proteins are involved in several processes, such as photosynthesis and in translation processes.

Included in the group of proteins with reduced accumulation at both time points were those involved in ribosome biogenesis (Table 3). The H/ACA ribonucleoprotein complex subunit 1 (H/ACA), that plays a central role in ribosomal RNA processing and ribosome biogenesis, was repressed to negative folds at 12 hpi (−4.0-fold). A 40S ribosomal protein subunit, RPS14, was also down accumulated at both time-points. RPS proteins are linked to protein synthesis. RPS14 has been described to be up regulated in watermelon when challenged with cucumber green mottle mosaic virus [52]. It is known that a repression of protein synthesis is usually associated with the selective translation of mRNA that encode proteins that are crucial for stress recovery, and thus cell survival. This translation repression reduces the energy toll on cells during a stress state [53].

A protease involved in cellular catabolic processes was also found to be repressed. The thiol protease aleurain-like isoform X1 is an amino and endopeptidase that hydrolyses proteins and, to our knowledge, it has not been described to have a role in plant–pathogen resistance or defense.

The HSP22, which was repressed at both time-points, is the only small HSP that is localized in the endoplasmic reticulum (ER) but its function is poorly known. Li and colleagues have reported that HSP22 may affect intracellular vesicle traffic of PIN proteins and its overexpression increases lateral root growth when seeds are under an auxin treatment [54].

The CP43 chlorophyll apoprotein from the photosystem II (PSII) was highly repressed in Regent upon inoculation with *P. viticola* pathogen. Its role involves chlorophyll binding and catalysis of the primary light-induced photochemical processes of the PSII. Its gene has been described to be down-regulated in *Vitis* upon drought stress [55] and its silencing promoted a higher accumulation of CMV virus in hot pepper [56]. The photosynthetic process is one of the most affected upon biotic stresses, responding to stress perception. To boost defense, plants reduce the resources allocated to growth thus resulting in a low photosynthesis capacity [57].

### 2.5. The DAPs Accumulated at Both Time Points of Grapevine–P. viticola Interaction Reflect Chloroplast Translation and Defense Response

Some proteins that were identified were up accumulated in both time-points (Table 4) are mostly defense-related proteins and related to translation in the chloroplast.

Two chloroplastic ribosomal proteins, 50S ribosomal protein L1 (RPL1) and 30S ribosomal protein S1 (RPS1), were identified to be up accumulated in grapevine Regent cultivar inoculated with *P. viticola*. The 50S and 30S subunits together form the plastid ribosome and they participate in the translation of chloroplastic mRNAs and photosynthesis [58]. RPS1 has been described to be crucial for optimal photosynthesis and growth performance since it is responsible for the synthesis of thylakoid membrane proteins [59]. Their role in photosynthesis performance in plant defense scenarios has already been reported. RPL1 was described to be accumulated in resistant rice cultivars inoculated with sugarcane mosaic virus and repressed in susceptible cultivars [60].

Matrix attachment region-binding filament-like protein 1 (MFP1) is a plant-specific protein that binds double-stranded DNA and its expression is higher in light conditions. This protein has been localized in the stroma side of the thylakoids [61] and has been related to defense responses in rice. In rice cell cultures elicited with chitooligosaccharides (COS) derived from fungi, this protein was found to be up accumulated, implying that the plasma membrane proteins that interpret fungal compounds play an important role in defense signal transduction during pathogen attack [62]. In a similar assay, with tomato plants elicited with COS and pectin-derived oligogalacturonides (OGA), this protein was also up accumulated [63]. The combination of COS-OGA was also applied to grapevine plants challenged with powdery mildew and the severity of symptoms was attenuated in grapes [64]. These observations suggest that plasma membrane receptors perceive pathogen attack and resistant cultivars adjust photosynthesis to boost energy production to counterattack.

A protein involved in peroxisome biogenesis was also present at both time-points in the Regent cultivar. Peroxisomes are crucial for photorespiration, H_2_O_2_ scavenging and turnover. This specific protein, peroxisome biogenesis protein 19-2 (PEX19-2), has been described to, together with PEX3, direct peroxisomal membrane proteins (PMPs) into the ER and the peroxisomal vesicle membrane [65]. Peroxisomes are rich in catalases that decompose H_2_O_2_ [58]. This might be a protection strategy of the Regent cultivar against ROS species.

Several defense-related proteins were also observed to be up accumulated. Protein P21, also known as osmotin, belongs to the PR-5 family, a family of pathogenesis-related proteins with sequence similarities to thaumatin. This protein has been extracted from different plants and shown antifungal activity against a broad range of plant pathogens [66]. It has also been related to the induce systemic resistance associated to beneficial fungi [67]. Interestingly, Monteiro and colleagues have shown that grapevine osmotin is able to block the growth of several grapevine pathogens such as *Uncinula necator*, *Phomopsis viticola*, and *Botrytis cinerea*, suggesting that this protein may play a role in grapevine defense to pathogen attacks [68].

A peptidyl-prolyl cis-trans isomerase (PPIase), from the cyclophilin family, was also up accumulated to threefold upon inoculation with *P. viticola*. These enzymes promote protein folding. PPIase members have roles in hormone signalling, protein trafficking, transcription, plant growth, immune system, and thus stress responses [69]. In wheat, cyclophilin family members have been described to be up regulated upon *Puccinia striiformis* infection, an obligate biotrophic fungi that causes wheat stripe rust disease [70]. Cyclophilin genes from potato (StCyP) and from pepper (CACYP1) have also been described to be involved in the response to *Fusarium solani* and to *Xanthomonas campestris* infections, respectively [71,72]. In another study, Park and colleagues purified and characterized a PPIase belonging to the FKBP family from Chinese cabbage and reported its antifungal activity against *Candida albicans, Botrytis cinerea, Rhizoctonia solani,* and *Trichoderma viride* [73]. In Arabidopsis, the disruption of a PPIase from the cyclophilin family (AtCYP20-3) resulted in enhanced susceptibility to necrotrophic fungi and oomycete infection [74].

Another three proteins were also identified: RAD23c, haloacid dehalogenase-like hydrolase domain-containing protein and fruit protein pKIWI502. These were all more accumulated in Regent challenged with *P. viticola* than in the control samples. The ubiquitin receptor RAD23c, a member of the RADIATION SENSITIVE23 family, binds to ubiquitin-conjugates and delivers them to the 26S proteasome [75]. It has been described that its silencing leads to a higher sensitivity towards *B. cinerea* in tomato [53]. The fruit protein from kiwi plant, pKIWI502, has been reported to be involved in the fruit development of this plant [76]. Interestingly, it has also been suggested that this protein takes part in PCD [77] and it was found to be up accumulated in resistant cowpea upon challenge with *Colletotrichum gloeosporioides* [78].

These studies are in line with the observed in the present study, where these proteins were up accumulated at both time points tested after inoculation with *P. viticola* pathogen.

## 3. Materials and Methods

### 3.1. Plant Material and Inoculation Experiments

*Vitis vinifera* cv Regent (ViVC number 4572) is a crossing line, bred for both *Plasmopara viticola* and *Erysiphe necator* resistance at Julius Kuhn Institute (JKI, Germany). It presents the resistance to *P. viticola* loci *Rpv3.1* and the resistance to *E. necator* loci 3 and 9 (REN3 and REN9), displaying a high degree of tolerance to both mildews [79].

*P. viticola* inoculations were carried out in greenhouse grown *Vitis vinifera* cv Regent plants, as previously described [26]. Briefly, a *P. viticola* inoculum was collected after an overnight incubation of symptomatic leaves from greenhouse infected plants in a moist chamber at room temperature. Sporangia were carefully collected by brushing the abaxial surfaces, dried and stored at −20 °C. Preceding inoculation, sporangia viability was confirmed by microscopic observations as described in [80]. A suspension containing 10^4^ sporangia mL^−1^ was used to spray the abaxial leaf surface, while controls were made by spraying the leaves with water (mock inoculations). After inoculation, plants were kept for 8 h in the dark at 25 °C and 99–100% relative humidity and then kept under greenhouse conditions during the inoculation time course. The third to fifth fully expanded leaves below the shoot apex were collected at 6 and 12 h post inoculation (hpi), immediately frozen in liquid nitrogen and stored at −80 °C. Three independent biological replicates were collected for each condition (inoculated and mock inoculated). Successful colonization of grapevine plants by *P. viticola* pathogen was confirmed by qPCR with primers for pathogen effectors (see supplementary information file).

### 3.2. Sample Preparation

Protein extraction was done using a phenol-based protocol according to [81], four biological replicates were extracted from each condition. Protein concentration was determined with a 2-D Quant Kit (GE Healthcare, Chicago, IL, USA) using bovine serum albumin (BSA; 2 mg/mL) as standard. Further purification was done with the Ettan 2D Clean-up kit (GE Healthcare, Chicago, IL, USA) according to manufacturer’s recommendations. The recovered precipitated protein was solubilized in 30 μL of labelling buffer and pH was adjusted to 8.5 using NaOH (100 mM).

Protein tryptic digestion was performed according to [82]. Shortly, disulfide bonds were reduced by DTT and cysteine residues alkylated with iodoacetamide. Samples were digested with trypsin (Promega) and the reaction was stopped by addition of 0.5% formic acid.

### 3.3. Liquid Chromatography Mass Spectrometry-Based Proteomics

To investigate the impact of *P. viticola* on *Vitis vinifera* cv Regent plants at several time-points after inoculation, we performed liquid chromatography coupled with mass spectrometry (LC-MS). A total of 2 µg of each digest was first separated by a nano-HPLC system (Proxeon, Odense, Denmark) and then the peptide mass spectra acquired using a Maxis Impact Q-TOF spectrometer (Bruker, Bremen, Germany). The peptides were first concentrated on a 100 μm ID 2 cm nanotrapping column (Proxeon, Odense, Denmark) and then loaded onto a 75 μm ID, 25 cm Acclaim PepMap nanoseparation column (Thermo Fischer Scientific, Waltham, MA, USA). The chromatography run using a 0.1% formic acid–acetonitrile gradient (2–30% in 120 min at a flow rate of 300 nL/min). The column was coupled to the mass spectrometer inlet through a Captive Spray ionization source (Bruker, Bremen, Germany). MS acquisition was set to cycles of MS, each followed by 3 cycles of MS/MS, with an intensity threshold for fragmentation of 2000 counts, and using a dynamic exclusion time of 2 min, with an automated precursor re-selection when a 3-fold increase in intensity was observed. Spectra were acquired on the range of 150-2200 Da. LC-MS/MS data were pre-processed using the Data Analysis 4.2 software (Bruker, Bremen, Germany). Data normalization, database searching, and protein identification were performed using MaxQuant software (v1.6.1.0) and protein quantification was determinate by MaxLFQ algorithm. Searches on a *Vitis vinifera* database retrieved from NCBI database (PRJNA33471, downloaded on May 2019, containing 41 208 protein sequences) included trypsin as digesting enzyme with a maximum of 2 missed cleavages; cysteine carbamidomethylation set as fixed modification and methionine oxidation as variable modification. The peptide mass tolerances of the first search and main search (recalibrated) were < 0.07 and 0.006 Da, respectively. The minimum peptide length was seven amino acids, and the maximum peptide mass was 4600 Da. Both peptides and proteins were filtered with a maximum false discovery rate (FDR) of 0.01. The match between runs feature with a matching window of 0.7 min and an alignment window of 20 min, was activated. Label-free quantitation (LFQ) calculations were performed separately in each parameter group containing similar cell loadings. All peptides were selected for protein quantification. Other unmentioned parameters were the MaxQuant default settings.

The mass spectrometry proteomics data was deposited to the ProteomeXchange Consortium via the PRIDE partner repository with the dataset identifier PXD 21613.

### 3.4. Differential Accumulation Analysis

MaxQuant “ProteinGroups” file was analyzed in R (R Core Team) with the R package “DEP” [83] for differential enrichment analysis of proteomics data. Proteins not present in at least 2 out of the 3 biological replicates of each experimental condition were removed. Background correction and data normalization were performed by variance stabilizing transformation [84]. Missing values were inputted using random sampling from a Gaussian distribution centered around a minimal value (“DEP” default) and intensities are converted to a log_2_ scale. Differential enrichment analysis was performed based on linear models and empirical Bayes statistics for each inoculated time-point and the respective control as described in [83]. Proteins with p-values lower than 0.05 were considered significant. The R code used is available at https://github.com/RuiNascimento/Vvinifera_Pviticola_first_hours_proteome.

### 3.5. GO Term Enrichment Analysis

In order to do a GO enrichment analysis, the NCBI gene ID for each protein ID was accessed with the R package “rentrez” [85] that provides an interface in R to NCBI’s Eutils API. The gene ID is used as reference for the GO terms search. To match the gene ID to GO terms in a fast and reliable way a SQLite annotation data package was created using a modified version of the popular AnnotationForge R package, adapted to work with plants genomes (https://github.com/RuiNascimento/AnnotationForge).

Biological Process and Molecular Function GO term enrichment analysis for each time point was done with the R package TopGO [86,87]. Only significant proteins (*p* value < 0.05) were selected and the test was run with the “classic” algorithm and “fisher” statistics. GO terms with *p* value < 0.05 and with 3 or more proteins differentially accumulated were considered significant. R code used in https://github.com/RuiNascimento/Vvinifera_Pviticola_first_hours_proteome.

Due to the hierarchical nature of Gene Ontologies we performed a semantic reduction of GO terms using the “rrvgo” R package, grouping similar terms based on their semantic similarity. For each time-point and both Biological Process and Molecular Function GO terms, similarity matrix were created using the “Rel” (Relevance) as the method and “org.At.tair.db” (*Arabidopsis thaliana*) as a reference database. Similarity matrix were reduced using a threshold of 0.7 and using −log(*p* value) as scores, meaning a lower *p* value equals a higher score.

## 4. Conclusions

In plant–pathogen interactions both organisms secrete molecules which will have a crucial role on how successful the infection will be. The plant will mainly secrete proteins that allow the build-up of a defense response while the pathogen will secrete effectors that will counterattack the plant defense strategy. In the case of grapevine–*P. viticola* pathosystem, both plant proteins that confer resistance as well as pathogen effectors are poorly known. This study interrogates the proteomic modulation of a highly tolerant grapevine hybrid cultivar by *P. viticola* in the first h of the interaction. This period is a crucial step in the infection process since it is when plant and pathogen first become in contact and the plant defense strategy begins.

Jones and Dangl proposed a zigzag model of interaction between plants and pathogens in 2006 but biological processes are usually plastic and different phases of the zigzag model could be observed simultaneously at a given time-point of analysis. In this study, effector-triggered susceptibility (ETS) and effector-triggered immunity (ETI) characteristic proteins are detected at both time-points; thus, these processes may be taking place between 6 and 12 hpi. ETS supresses the PAMP-triggered immunity (PTI) at the first level of molecular co-evolution between host and pathogen. This occurs through pathogen effectors suppression of plant immunity by direct interaction with host defense-associated proteins [2].

The increase in accumulation of proteins related to plant stress defense might be an indication that at 12 hpi, the *V. vinifera* Regent plants are counterattacking the *P. viticola* pathogen in an ETI responsive manner. In contrast, we also observed the repression of plant defense proteins, such as serine/threonine kinase protein, at 12 hpi. The observations made in here point to a plastic response from both grapevine and *P. viticola* in their interaction. To our knowledge, this is the first time that early infection time-points are reported for this pathosystem.

Further studies need to be conducted to increase the knowledge in grapevine resistance towards *P. viticola*. The proteomic modulation of other cultivars, with different degrees of resistance or tolerance, must be analyzed so that a comprehensive knowledge is generated regarding grapevine defense strategies towards oomycetes.

## Figures and Tables

**Table 1:** Proteins up accumulated at 6 hpi and repressed at 12 hpi in grapevine–*P. viticola* interaction.

Protein Name	Protein Code (NCBI Database)	log_2_ (FC)	
		6 hpi	12 hpi
PREDICTED: 50S ribosomal protein L3, chloroplastic (*Vitis vinifera*)	XP_002271466.1	2.4	−2.3
PREDICTED: nascent polypeptide-associated complex subunit alpha-like protein 2 (*Vitis vinifera*)	XP_003634163.1	3.4	−1.6
PREDICTED: nascent polypeptide-associated complex subunit alpha-like protein 1 (*Vitis vinifera*)	XP_003632619.1	1.3	−2.2
PREDICTED: major allergen Pru ar 1 (*Vitis vinifera*)	XP_002273790.2	3.0	−1.5
serine/threonine kinase-like (*Vitis vinifera*)	NP_001268124.1	1.5	−2.1

**Table 2:** Proteins repressed at 6 hpi and up accumulated at 12 hpi in grapevine–*P. viticola* interaction.

Protein Name	Protein Code (NCBI Database)	log_2_ (FC)	
		6 hpi	12 hpi
PREDICTED: aminomethyltransferase, mitochondrial (*Vitis vinifera*)	XP_002272701.1	−1.7	5.4
PREDICTED: elongation factor 1-alpha (*Vitis vinifera*)	XP_002277159.1	−3.1	2.6
PREDICTED: actin-depolymerizing factor 2 (*Vitis vinifera*)	XP_002284292.1	−4.2	2.9
PREDICTED: serine hydroxymethyltransferase, mitochondrial (*Vitis vinifera*)	XP_010646402.1	−4.3	2.3
PREDICTED: heat shock 70 kDa protein, mitochondrial isoform X1 (*Vitis vinifera*)	XP_002263457.1	−2.1	2.1
PREDICTED: glyceraldehyde-3-phosphate dehydrogenase B, chloroplastic (*Vitis vinifera*)	XP_002273754.1	−0.9	2.2

**Table 3:** Proteins repressed at 6 hpi and 12 hpi in grapevine–*P. viticola* interaction.

Protein Name	Protein Code (NCBI Database)	log_2_ (FC)	
		6 hpi	12 hpi
PREDICTED: 40S ribosomal protein S14 (*Vitis vinifera*)	XP_002274381.1	−1.7	−3.3
PREDICTED: 22.0 kDa class IV heat shock protein (*Vitis vinifera*)	XP_002263376.1	−3.2	−2.8
PREDICTED: H/ACA ribonucleoprotein complex subunit 1 (*Vitis vinifera*)	XP_002277849.1	−2.1	−4.0
photosystem II CP43 chlorophyll apoprotein (chloroplast) (*Vitis vinifera*)	ABE47530.1	−2.8	−4.1
PREDICTED: thiol protease aleurain-like isoform X1 (*Vitis vinifera*)	XP_002278624.1	−6.6	−1.4

**Table 4:** Proteins induced at both time-points in grapevine–*P. viticola* interaction.

Protein Name	Protein Code (NCBI Database)	log_2_ (FC)	
		6 hpi	12 hpi
PREDICTED: 50S ribosomal protein L1, chloroplastic (*Vitis vinifera*)	XP_002274498.1	2.0	1.3
PREDICTED: 30S ribosomal protein S1, chloroplastic (*Vitis vinifera*)	XP_002280604.1	1.7	2.4
PREDICTED: MAR-binding filament-like protein 1-1 isoform X1 (*Vitis vinifera*)	XP_002284745.2	3.9	2.2
PREDICTED: peroxisome biogenesis protein 19-2 (*Vitis vinifera*)	XP_002269360.1	2.3	3.0
PREDICTED: protein P21 (*Vitis vinifera*)	XP_002283030.1	2.3	3.0
PREDICTED: peptidyl-prolyl cis-trans isomerase (*Vitis vinifera*)	XP_002273421.2	2.1	3.4
PREDICTED: ubiquitin receptor RAD23c (*Vitis vinifera*)	XP_002283656.1	2.8	2.4
PREDICTED: fruit protein pKIWI502 (*Vitis vinifera*)	XP_002283966.1	2.0	3.9
PREDICTED: haloacid dehalogenase-like hydrolase domain-containing protein At3g48420 (Vitis vinifera)	XP_002277650.1	2.7	6.0

## Supplementary Materials

The following are available online at https://www.mdpi.com/2223-7747/9/11/1498/s1, Table S1: List of *V. vinifera* differentially accumulated proteins at 6 and 12 hpi., Table S2: List of significant *V. vinifera* proteins differentially accumulated at 6 hpi (*p* value < 0.05)., Table S3: List of significant *V. vinifera* proteins differentially accumulated at 12 hpi (*p* value < 0.05).
